# Efficacy of tolvaptan on the short and mid-term prognosis in elderly patients with acute heart failure coexisting with oliguria: A retrospective cohort study

**DOI:** 10.3389/fcvm.2022.1075631

**Published:** 2023-01-09

**Authors:** Yang Liu, Yabin Zhang, Hongyu Chen, Jiahui Zhao, Qiang Ma, Guang Yang, Xiaohua Wang, Zhen Wu, Jiebin Hou, Qingli Cheng, Qiangguo Ao

**Affiliations:** Department of Nephrology, The Second Medical Center of Chinese PLA General Hospital, National Clinical Research Centre for Geriatric Diseases, Beijing, China

**Keywords:** tolvaptan, acute heart failure (AHF), oliguria, elderly, prognosis

## Abstract

**Background:**

In patients with acute heart failure (AHF) coexisting with oliguria, high doses of loop diuretics are often ineffective in increasing urine output and may adversely affect the patient's prognosis, especially in elderly patients. We investigated the efficacy of adding tolvaptan (TLV) on improving the prognosis in elderly patients with AHF coexisting with oliguria.

**Methods:**

All data for this retrospective cohort study were extracted from the electronic medical record system of the Second Medical Center of Chinese PLA General Hospital from January 2018 to December 2020. Patients diagnosed with AHF coexisting with oliguria were enrolled in this study and were divided into TLV and non-TLV groups based on the use of TLV. The primary outcome was all-cause mortality at 7 and 90-day. The secondary outcomes were the remission of AHF within 7 and 30 days or continued progression of AHF, and new-onset chronic kidney disease (CKD) after 90 days. Cox proportional hazards regression was used to assess the relationships between all-cause mortality and diuretic regimens, demographics, laboratory parameters, comorbidities, and medications.

**Results:**

A total of 308 patients met the study criteria for the final statistical analysis, and they had a median age of 91 years (88, 95). The results showed that the addition of TLV was associated with a decreased risk of the 7 and 90-day all-cause mortality in patients with AHF with oliguria [adjusted HR, 95% CI: 0.60 (0.37, 0.98), *p* = 0.042; 0.56 (0.41, 0.75), *p* < 0.001, respectively]. Adding TLV significantly increased urine output and decreased N-terminal pro-B-type natriuretic peptide (NT-proBNP) levels in 7 days, and alleviated the progression of AHF within 30 days. There were no statistically significant differences between the patients with or without TLV in terms of the occurrence of hypernatremia, the development of hepatic impairment within 30 days, and new-onset CKD after 90 days.

**Conclusions:**

This study demonstrated that the addition of TLV was clinically effective in increasing urine output, and had favorable effects on alleviating AHF progression and may reduce the risk of all-cause mortality at 7 and 90-day in elderly patients with AHF with oliguria, and TLV had a good safety profile.

**Trial registration:**

http://www.chictr.org.cn/showprojen.aspx?proj=148046, identifier: ChiCTR2200055518.

## Introduction

Acute heart failure (AHF) is a frequent cause of hospitalization for patients with heart disease, and patients with AHF, especially older patients, are at high risk of rehospitalization and have worse short- and long-term survival and mortality rates (1, 2). As the population of aging adults increase, the incidence of AHF and its associated health and economic burdens are expected to increase further (3, 4).

Achievement of volume reduction or fluid removal was fundamental to the treatment of AHF. As a result, loop diuretics, such as furosemide, are widely used in high doses in the clinical treatment of AHF (5, 6). This is accompanied by diuretic resistance, activation of the renin-angiotensin-aldosterone system (RAAS), impairment of renal function, and even the occurrence of acute kidney injury (AKI). Diuretic resistance is defined as an impaired sensitivity to diuretics resulting in reduced natriuresis and diuresis limiting the possibility of achieving euvolemia (7). Diuretic resistance is different from oliguria, and patients with diuretic resistance may not meet the criteria of oliguria. The presence of oliguria may mean more severe diuretic resistance which leads to worse prognosis in patients with AHF, especially in elderly patients (8–10). Currently, there is no approved strategy for treating this high-risk subgroup. TLV combined with loop diuretic therapy may have favorable outcomes (11).

Tolvaptan (TLV) is a selective vasopressin V2-receptor antagonist that competes with the antidiuretic hormone vasopressin for the V2 receptor in the renal collecting duct, resulting in inhibition of water resorption by the collecting ducts of the kidney, and increased excretion of free water (12). TLV increases urine output without inducing intravascular volume depletion or activation of the RAAS (13). Moreover, TLV reduces the use of loop diuretics, even in patients who are resistant to loop diuretics, and is clinically effective in relieving congestion and improving renal function in AHF patients (14).

TLV combined with loop diuretics has been shown to be effective in the treatment of diuretic-resistant AHF patients (6, 15, 16). However, there are few reports on whether TLV is equally effective in elderly patients with AHF and oliguria (10, 17). For this reason, in this study, we examined the efficacy and safety of TLV in elderly patients with AHF and oliguria.

## Methods

### Study design and population

This retrospective, single-center study, was conducted in the Second Medical Center of Chinese PLA General Hospital in Beijing, China. This study was approved by the Ethics Committee of Chinese PLA General Hospital (No. S2022-342-01). Written informed consent was not required for this study because the data were collected anonymously. This study was registered at http://www.chictr.org.cn/showprojen.aspx?proj=148046 (ChiCTR2200055518).

**Figure 1:**
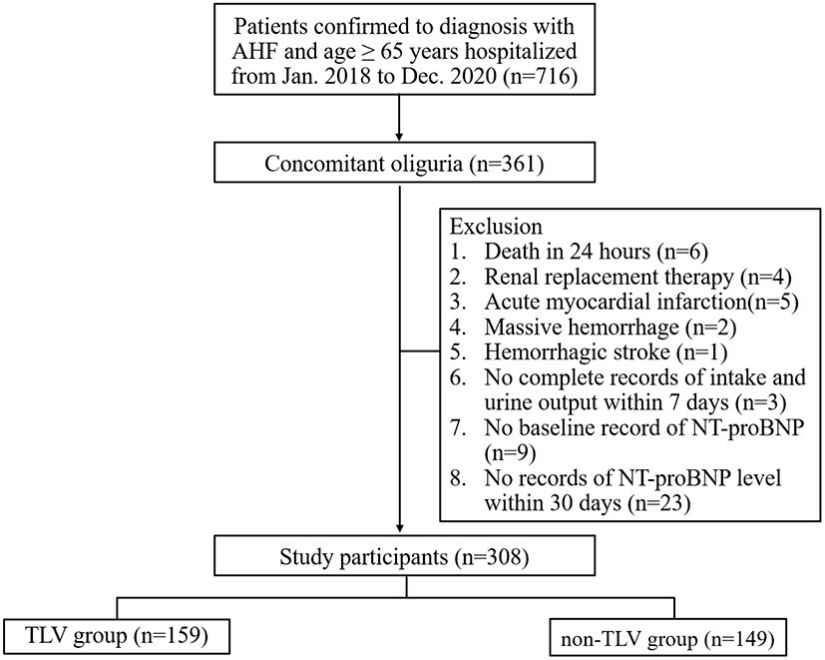
Flow chart of patient enrollment in this study. NT-proBNP, N-terminal pro-B-type natriuretic peptide.

The patients meeting all of the following criteria were included: (1) age ≥65 years; (2))diagnosis of AHF, as determined by echocardiography showing an ejection fraction <50% and/or N-terminal pro-B-type natriuretic peptide (NT-proBNP) ≥1800 pg/ml (18, 19), and ≥1.5-fold increase in NT-proBNP level from baseline (the baseline level of NT-proBNP defined as the most recent NT-proBNP value measured between 7 and 365 days prior to the onset of oliguria in AHF patients); (3) coexisting oliguria (set as the starting point of this study). In this study, oliguria was defined as a urine output < 0.5 ml/(kg·h) for more than 12 h (20); (4) If patients treated with TLV, the duration of TLV treatment was more than 30 days or until the patient died. The patients meeting at least one of the following criteria were excluded: (1) death within 24 h of the onset of oliguria; (2) accepted renal replacement therapy (RRT), implantation of a mechanical circulatory support (MCS) or ICD, CRT-D; (3) obstructive and non-obstructive type of hypertrophic cardiomyopathy, restrictive cardiomyopathy, cardiac amyloidosis, significant valvular heart disease, acute myocardial infarction, cardiac arrest, massive hemorrhage (bleeding of more than 1000 ml/24 h within 7 days), hemorrhagic stroke; (4) no complete intake and urine volume records after the onset of oliguria; (5) no baseline record of NT-proBNP; (6) no NT-proBNP level available within 7 days and 30 days after the occurrence of oliguria; and 7) missing data or clinically unreliable data. The flow chart of this study is shown in Figure 1.

All patients received loop diuretics and standard AHF treatment regimens under the supervision of their physicians. The average intake or urine output was calculated as the average daily intake or urine output over the 7 days after the onset of oliguria. Daily doses of loop diuretics are represented by furosemide, and doses of other loop diuretics were converted into equivalent furosemide doses using the formula: 1 mg bumetanide equivalent to 20 mg torsemide, and equivalent to 40 mg furosemide (21, 22). Depending on whether TLV was used, the patients were divided into two groups: the TLV group and the non-TLV group. The patients in the non-TLV group received 20 to 400 mg equivalent dose of furosemide daily for the treatment of oliguria. The patients in the TLV group received an additional 15 to 45 mg of TLV daily on top of the non-TLV group. TLV was added within 72 h of the onset of oliguria in AHF patients, and the duration of TLV treatment was more than 30 days or until the patient died. The patients were divided into six groups based on the use of equivalent doses of furosemide daily and the additional use of TLV: Group A1: <100 mg/d equivalent doses of furosemide plus TLV; Group A0: <100 mg/d equivalent doses of furosemide only; Group B1: ≥100 and <200 mg/d equivalent doses of furosemide plus TLV; Group B0: ≥100 and <200 mg/d equivalent doses of furosemide only; Group C1: ≥200 mg/d equivalent doses of furosemide plus TLV; and Group C0: ≥200 mg/d equivalent doses of furosemide only.

### Clinical data and outcomes

All patients were male because our hospital is a military hospital. Age, systolic blood pressure (SBP), diastolic blood pressure (DBP), mean arterial pressure (MAP), intake, urine output, laboratory parameters, comorbidities, medications, and outcome data (death or not) were extracted from the Second Medical Center of Chinese PLA General Hospital electronic medical record system from January 2018 to December 2020. All data were entered into a computer database and were cross-checked by two independent physicians (Yang Liu and Yabin Zhang). The principal investigator (Qiangguo Ao) regularly performed randomized group reviews and blinded reviews of the groups.

The primary outcome was all-cause mortality at 7 and 90-day. The secondary outcomes were reversal or continued progression of AHF within 7 and 30 days, and new-onset CKD after 90 days. In this study, reversal of AHF within 7 or 30 days was defined as the NT-proBNP level persistently decreased above 1.5 times the baseline level. Continued progressive AHF was defined as the NT-proBNP level persistently elevated above 1.5 times the baseline level. The new-onset CKD was defined as the patients with eGFR < 60 ml/min/1.73 m^2^ after 90 days, expect the history of CKD.

### Statistical analysis

We used SPSS software (Version 26.0) to perform data analysis in this study. We evaluated the normality of the distribution of variables using the Kolmogorov–Smirnov test. Continuous variables were reported as the means with standard deviations or medians with interquartile ranges according to normality. The difference between normally distributed continuous variables was tested by the *t*-test or ANOVA, and non-normally distributed data were tested by the Mann–Whitney U test or Kruskal–Wallis test. Categorical variables were presented as numbers (%) and were compared with the chi-square test and Fisher's exact test. The one-way ordered classified variables were compared with the rank sum test. Survival was analyzed by the Kaplan–Meier method. The log rank method was used to compare the survival of two or more groups of patients. Prognostic analysis was performed using Cox regression. All tests were two sided, and a *p-*value < 0.05 was considered statistically significant.

## Results

### Patient characteristics

From January 2018 to December 2020, 716 patients aged ≥ 65 years were diagnosed with AHF at the Second Medical Center of Chinese PLA General Hospital. Among them, 361 patients developed oliguria. A total of 308 patients were enrolled in this study according to the inclusion and exclusion criteria. Of these, 159 (51.6%) patients belonged to the TLV group with a median age of 92 years (88, 95), and 149 (48.4%) patients belonged to the non-TLV group with a median age of 91 years (87, 94). The median MAP level was 77 mmHg (69, 86), and the median eGFR was 34.9 ml/min/1.73 m^2^ (24.5, 49.2) in all enrolled patients. There was no significant difference in baseline renal function between the two groups [TLV group: eGFR 35.9 (25.2, 50.8) vs. non-TLV group: eGFR 34.7 (23.9, 47.2), ml/min/1.73 m^2^, *p* = 0.712]. Compared with the non-TLV group, the patients in the TLV group had lower BUN levels [17.50 (11.90, 23.50) vs. 20.39 (12.83, 31.54), mmol/L, *p* = 0.024] and were more likely to be taking spironolactone [59 (37.1%) vs. 38 (25.5%), *p* = 0.028], and the difference was statistically significant. Table 1 showed that there were no significant differences between the two groups regarding the proportion of patients with comorbidities (hypertension, diabetes mellitus, chronic kidney disease, coronary artery disease, atrial fibrillation), the relevant laboratory parameters (hemoglobin, serum albumin, and sodium), and the proportion of patients who took concomitant medications (hydrochlorothiazide, ARB/ACEI, β-blocker). The baseline characteristics of the patients in each group are shown in Table 1.

**Table 1:** Baseline characteristics of the study population.

**Characteristics**	**Total (*n =* 308)**	**Tolvaptan^#^(*n =* 159)**	**Non-tolvaptan (*n =* 149)**	***p*-value**
**Demographics**				
Age, years	91 (88, 95)	92 (88, 95)	91 (87,94)	0.116
Sex, male	308 (100%)	159 (100%)	149 (100%)	
BMI, kg/m^2^	23.4 (21.3, 25.6)	23.4 (20.9, 25.6)	23.4 (21.5, 25.7)	1.00
SBP, mmHg	112 (101, 126)	112 (102, 127)	112 (100, 125)	0.544
DBP, mmHg	58 (52, 66)	59 (52, 68)	58 (51, 65)	0.304
MAP, mmHg	77 (69, 86)	77 (70, 87)	76 (68, 84)	0.329
**Comorbidities**, ***n*** **(%)**				
Hypertension	247 (80.2%)	133 (83.6%)	114 (76.5%)	0.116
Diabetes mellitus	166 (53.9%)	85 (53.5%)	81 (54.4%)	0.874
CAD	258 (83.8%)	135 (84.9%)	123 (82.6%)	0.575
Atrial fibrillation	115 (37.3%)	59 (37.1%)	56 (37.6%)	0.931
EF				0.210
EF > 50%	247 (80.2%)	124 (78.0%)	123 (82.6%)	
40% ≤ EF ≤ 50%	37 (12.0%)	24 (15.1%)	13 (8.7%)	
EF < 40%	24 (7.8%)	11 (6.9%)	13 (8.7%)	
CKD	133 (43.2%)	68 (42.8%)	65 (43.6%)	0.879
Hyperlipidemia	142 (46.1%)	71 (44.7%)	71 (47.7%)	0.598
Hyperuricemia	216 (70.1%)	118 (74.2%)	98 (65.8%)	0.106
Stroke	159 (51.6%)	89 (56.0%)	70 (47.0%)	0.114
COPD	118 (38.3%)	57 (35.8%)	61 (40.9%)	0.358
Non-invasive ventilation	167 (54.2%)	81 (50.9%)	86 (57.7%)	0.233
Infection^*^	143 (46.4%)	75 (47.2%)	68 (45.6%)	0.788
History of cancer	136 (44.2%)	55 (34.6%)	81 (54.4%)	0.001
Abnormal liver function	130 (42.2%)	67 (42.1%)	63 (42.3%)	0.980
**Medications**, ***n*** **(%)**				
Doses of furosemide, mg	308 (100%)	159 (100%)	149 (100%)	
<100 mg/d	174 (56.5%)	104 (59.8%)	70 (40.2%)	
≥100 mg/d and <200 mg/d	63 (20.5%)	32 (50.8%)	31 (49.2%)	
≥200 mg/d	71 (23.1%)	23 (32.4%)	48 (67.6%)	
Spironolactone	97 (31.5%)	59 (37.1%)	38 (25.5%)	0.028
Hydrochlorothiazide	44 (14.3%)	25 (15.7%)	19 (12.8%)	0.456
ARB/ACEI	97 (31.5%)	52 (32.7%)	45 (30.2%)	0.636
β-Blockers	150 (48.7%)	79 (49.7%)	71 (47.7%)	0.721
Statin	121 (39.3%)	66 (41.5%)	55 (36.9%)	0.409
Nitrates	165 (53.6%)	98 (61.6%)	67 (45.0%)	0.003
Digoxin	34 (11.0%)	16 (10.1%)	18 (12.1%)	0.590
**Laboratory parameters**				
Hemoglobin, g/L	100 (85, 114)	101 (88, 116)	98 (83, 113)	0.161
Albumin, g/L	31.7 (28.6, 35.4)	32.2 (28.8, 35.4)	30.9 (28.1, 35.4)	0.142
Scr, mmol/L	151 (113, 198)	151 (109,193)	151 (116,201)	0.601
eGFR, ml/min/1.73 m^2^	34.9 (24.5, 49.2)	35.9 (25.2, 50.8)	34.7 (23.9, 47.2)	0.712
BUN, mmol/L	18.4 (12.5, 27.4)	17.5 (11.9, 23.5)	20.4 (12.8, 31.5)	0.024
Sodium, mmol/L	141 (136, 147)	140 (136, 147)	141 (137, 147)	0.349
Potassium, mmol/L	4.4 (4.0, 4.8)	4.4 (4.0, 4.8)	4.4 (3.9, 4.8)	0.714
TC, mmol/L	3.6 (2.9, 4.4)	3.6 (3.0, 4.4)	3.5 (2.9, 4.4)	0.489
TG, mmol/L	1.4 (0.9, 2.2)	1.2 (0.9, 2.1)	1.5 (1.0, 2.4)	0.074
HDL, mmol/L	0.82 (0.6, 1.1)	0.8 (0.6, 1.2)	0.8 (0.6, 1.1)	0.190
LDL, mmol/L	2.0 (1.5, 2.6)	2.1 (1.6, 2.5)	2.0 (1.4, 2.6)	0.299
Serum uric acid, mmol/L	406 (309, 525)	405 (304, 525)	407 (320, 528)	0.589
Baseline NT-proBNP	1,176 (470, 2986)	1,555 (490, 3283)	967 (439, 2755)	0.181

BMI, body mass index; SBP, systolic blood pressure; DBP, diastolic blood pressure; MAP, mean arterial pressure; Scr, Serum creatinine; eGFR, estimated glomerular filtration rate; TC, cholesterol; TG, triglyceride; HDL, high density lipoprotein cholesterol; LDL low density lipoprotein cholesterol; CAD, coronary atherosclerotic heart disease; EF, ejection fraction; CKD, chronic kidney disease; COPD, chronic obstructive pulmonary disease; ARB/ACEI, angiotensin receptor blocker /angiotensin-converting enzyme inhibitor.

* Infection including: respiratory tract infection, gastrointestinal infection, urinary tract infection, skin and soft tissue infection, and fever of unknown origin.

# Tolvaptan dose and duration: 15 to 45 mg/d, more than 30 days or until death.

### Changes in urine output and NT-proBNP level

There was no difference in the average volume intake between the TLV and non-TLV groups in 7 days. The addition of TLV significantly increased the median urine output of the patients in 7 days, and the difference between the two groups was statistically significant. The patients in the TLV group showed a gradual decrease in their NT-proBNP levels in 7 and 30 days after TLV administration. However, the patients in the non-TLV group showed a gradual increase from baseline level of NT-proBNP to the 30 days level of NT-proBNP. Compared with the non-TLV group, the TLV group had significantly lower NT-proBNP levels in 30 days, and the ratio of NT-proBNP to baseline NT-proBNP was significantly lower (Table 2).

**Table 2:** Changes of urine output and NT-proBNP level.

**Characteristics**	**Tolvaptan**	**Non-tolvaptan**	***p*-value**
Number of patients	159	149	
Average intake in 7 days, L	2.44 (2.17, 2.72)	2.32(1.97, 2.77)	0.086
Average urine output in 7 days, L	1.45 (1.11, 1.76)	0.85 (0.39, 1.21)	0.001
Baseline NT-proBNP level, pg/ml	1,554 (490, 3,283)	967 (439, 2,755)	0.181
NT-proBNP level in 0 day, pg/ml	5,538 (2,091, 1,2581)	4,369 (1,885, 9,981)	0.084
NT-proBNP level in 7 days, pg/ml	4,035 (1,572, 11432)	4,704 (1,978, 10,065)	0.771
NT-proBNP level in 30 days, pg/ml	3,108 (1,157, 7,821)	6,307 (1,407, 15,303)	0.007
NT-proBNP level in 7 days / Baseline NT-proBNP level	1.42 (0.26, 5.60)	2.30 (0.52, 9.20)	0.063
NT-proBNP level in 30 days / Baseline NT-proBNP level	0.81 (−0.02, 3.92)	2.86 (0.22, 12.39)	0.001

NT-proBNP, N-terminal pro-B-type natriuretic peptide.

### Cox proportional hazards regression analysis

**Figure 2:**
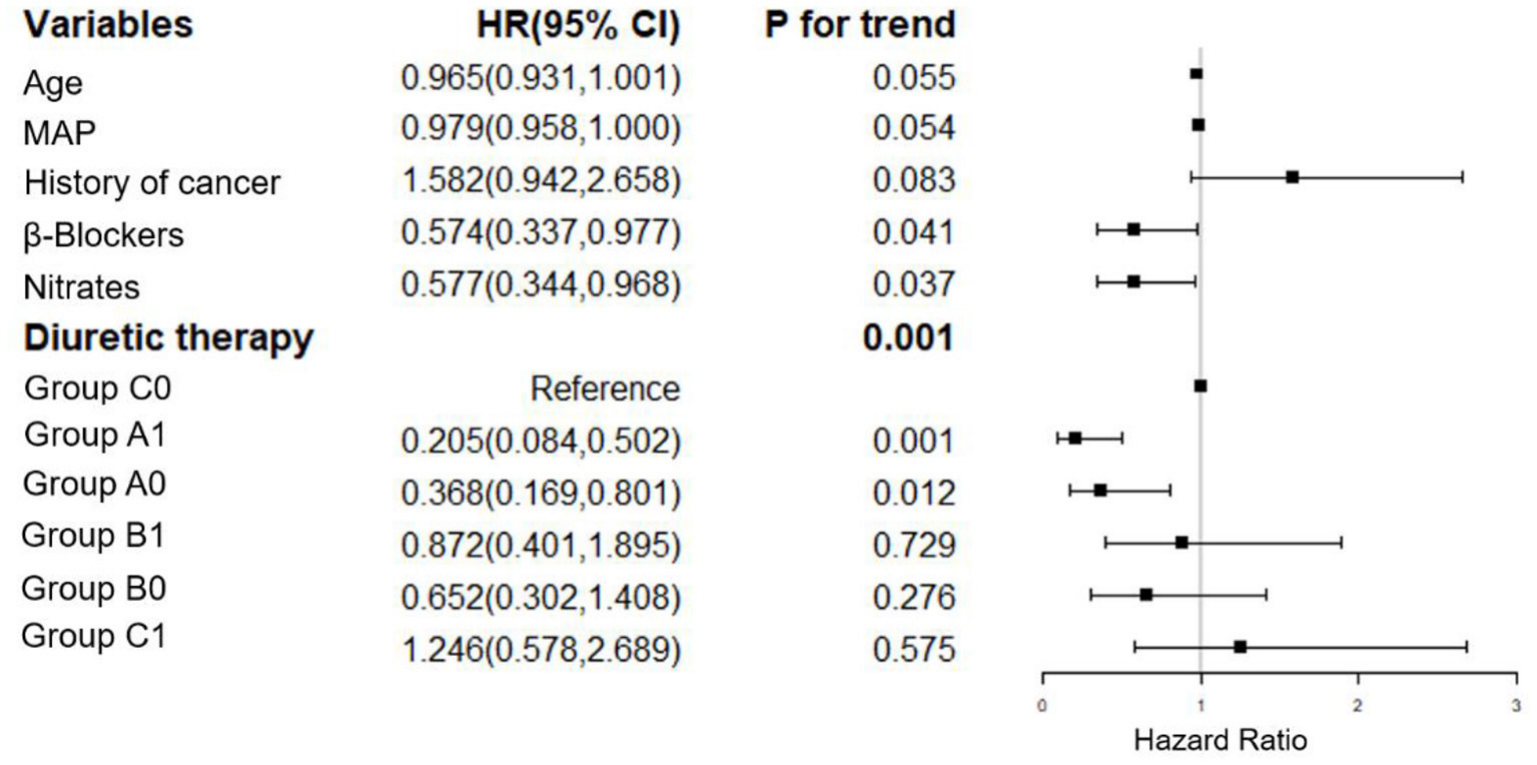
Cox regression analysis of the associations between 7-day cumulative mortality and the clinical findings. MAP, mean arterial pressure; Group A1: <100 mg/d equivalent doses of furosemide plus TLV; Group A0: <100 mg/d equivalent doses of furosemide only; Group B1: ≥100 and <200 mg/d equivalent doses of furosemide plus TLV; Group B0: ≥100 and <200 mg/d equivalent doses of furosemide only; Group C1: ≥200 mg/d equivalent doses of furosemide plus TLV; Group C0: ≥200 mg/d equivalent doses of furosemide only.

**Figure 3:**
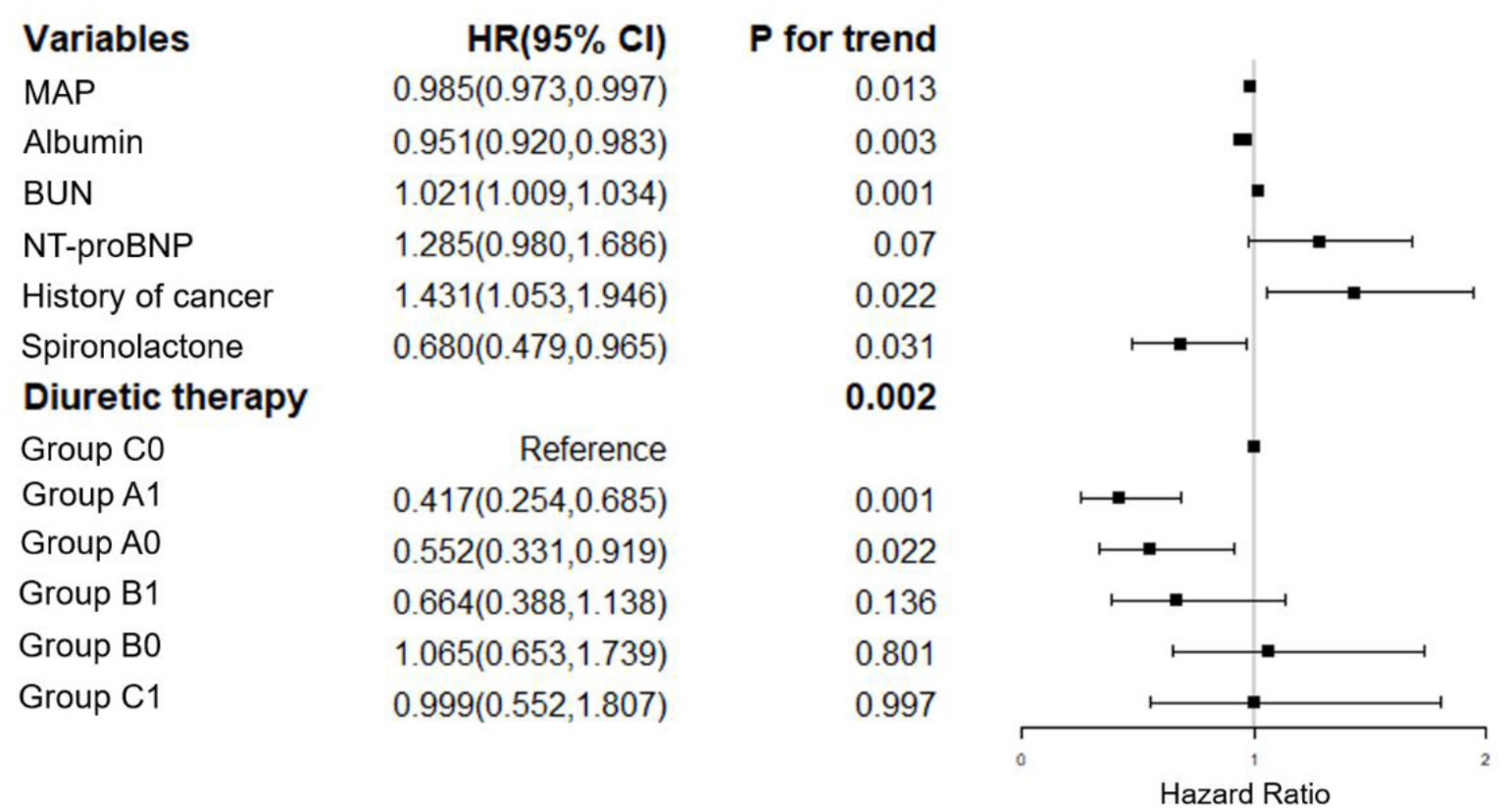
Cox regression analysis of the associations between 90-day cumulative mortality and the clinical findings. MAP, mean arterial pressure; NT-proBNP, N-terminal pro-B-type natriuretic peptide. Group A1: <100 mg/d equivalent doses of furosemide plus TLV; Group A0: <100 mg/d equivalent doses of furosemide only; Group B1: ≥100 and <200 mg/d equivalent doses of furosemide plus TLV; Group B0: ≥100 and <200 mg/d equivalent doses of furosemide only; Group C1: ≥200 mg/d equivalent doses of furosemide plus TLV; Group C0: ≥200 mg/d equivalent doses of furosemide only.

We have adjusted regarding demographics (BMI), comorbidities (diabetes mellitus, coronary artery disease, atrial fibrillation, EF, hyperlipidemia, hyperuricemia, stroke, COPD, non-invasive ventilation and infection), laboratory parameters (hemoglobin, albumin, eGFR, BUN, sodium, potassium, baseline NT-proBNP), and medications (spironolactone, hydrochlorothiazide, ARB/ACEI, statin, digoxin) for multivariate analysis of 7-day mortality (Figure 2). We have adjusted regarding demographics (age, BMI), comorbidities (diabetes mellitus, coronary artery disease, atrial fibrillation, EF, hyperlipidemia, hyperuricemia, stroke, COPD, non-invasive ventilation and infection), laboratory parameters (hemoglobin, eGFR, sodium, potassium, baseline NT-proBNP), and medications (hydrochlorothiazide, ARB/ACEI, β-Blockers, statin, digoxin) for the multivariate analysis of 90-day mortality (Figure 3). There were significant protective factors of β-blocker (*p* = 0.041) and nitrate (*p* = 0.037) use for 7-day all-cause mortality. The levels of MAP (*p* = 0.013), albumin (*p* = 0.003), and the use of spironolactone (*p* = 0.031) were significant protective factors for 90-day all-cause mortality. BUN level (*p* = 0.001) and cancer history (*p* = 0.022) were associated with an increased risk of 90-day all-cause mortality. The use of <100 mg equivalent furosemide daily only (Group A0) or the addition of TLV on this basis (Group A1) were protective factors for both 7-day all-cause mortality and 90-day all-cause mortality (Figures 2, 3).

### Primary outcome and subgroup analysis

**Figure 4:**
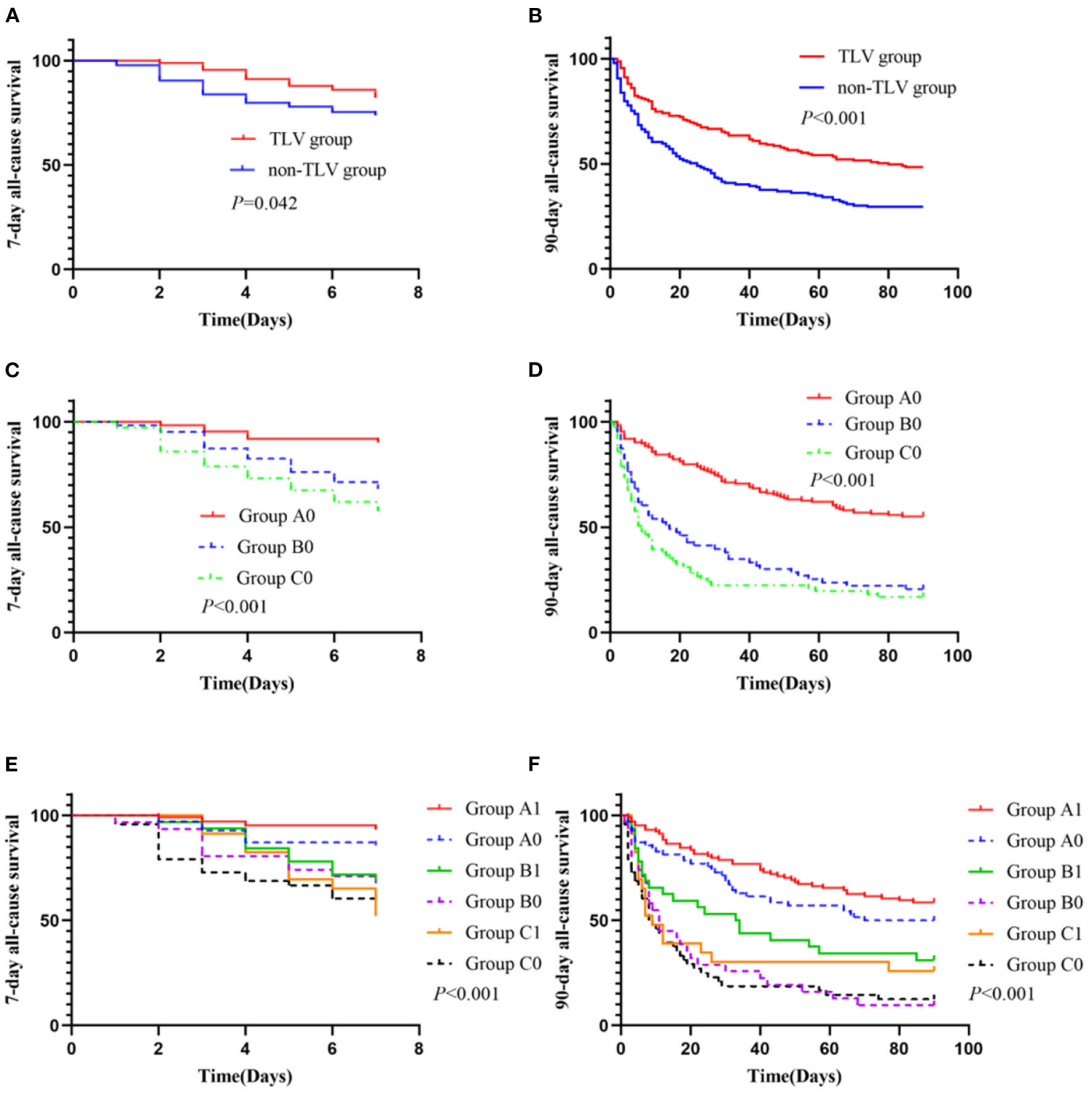
The primary outcome and subgroup analysis. Group A1 (*n* = 104): <100 mg/d equivalent doses of furosemide plus TLV; Group A0 (*n* = 70): <100 mg/d equivalent doses of furosemide only; Group B1 (*n* = 32): ≥100 and <200 mg/d equivalent doses of furosemide plus TLV; Group B0 (*n* = 31): ≥100 and <200 mg/d equivalent doses of furosemide only; Group C1 (*n* = 23): ≥200 mg/d equivalent doses of furosemide plus TLV; Group C0 (*n* = 48): ≥200 mg/d equivalent doses of furosemide only. **(C)** Subgroup comparison: Group A0 vs. Group B0, *p* < 0.001; Group A0 vs. Group C0, *p* < 0.001; Group B0 vs. Group C0, *p* = 0.179. **(D)** Subgroup comparison: Group A0 vs. Group B0, *p* < 0.001; Group A0 vs. Group C0, *p* < 0.001; Group B0 vs. Group C0, *p* = 0.190. **(E)** Subgroup comparison: Group A0 vs. Group A1, *p* = 0.096; Group B0 vs. Group B1, *p* = 0.037; Group C0 vs. Group C1, *p* = 0.848; Group A1 vs. Group B1, *p* = 0.057; Group A1 vs. Group C1, *p* < 0.001; Group B1 vs. Group C1, *p* = 0.264; **(F)** Subgroup comparison: Group A0 vs. Group A1, *p* = 0.180; Group B0 vs. Group B1, *p* = 0.033; Group C0 vs. Group C1, *p* = 0.241; Group A1 vs. Group B1, *p* = 0.001; Group A1 vs. Group C1, *p* < 0.001; Group B1 vs. Group C1, *p* = 0.416.

The addition of TLV had favorable effects on improving the 7 and 90-day all-cause mortality in elderly patients with AHF combined with oliguria [adjusted HR, 95% CI: 0.60 (0.37, 0.98), *p* = 0.042; 0.56 (0.41, 0.75), *p* < 0.001, respectively; Figures 4A, B]. A subgroup analysis showed that the use of more than 100 mg equivalent doses of furosemide daily was associated with an increased risk of all-cause mortality at 7 and 90-day [group A0 vs. group B0, *p* < 0.001; group A0 vs. group C0, *p* < 0.001; Figures 4E, F]. The difference in all-cause mortality between group B0 (patients on ≥100 and <200 mg/d equivalent doses of furosemide only) and group C0 (patients on ≥200 mg/d equivalent doses of furosemide only) was not statistically significant. However, the addition of TLV was associated with a decreased risk of all-cause mortality at 7 and 90-day when using ≥100 and <200 mg/d equivalent doses of furosemide.

### Secondary outcomes and safety analysis

The addition of TLV significantly decreased the NT-proBNP levels and alleviated the progression of AHF within 30 days. However, the use of TLV did not have a statistically significant effect on the alleviation of AHF progression within 7 days and new-onset CKD after 90 days. Compared with the non-TLV group, there were no statistically significant differences in hypernatremia, hyponatremia, or hepatic impairment within 7 and 30 days in the TLV group (Table 3).

**Table 3:** Safety analysis.

**Characteristics**	**Tolvaptan**	**Non-tolvaptan**	***p*-value**
Number of patients	159	149	
AHF progression within 7 days	109 (68.6%)	114 (76.5%)	0.118
AHF progression within 30 days	91 (57.2%)	103 (69.1%)	0.031
New-onset CKD after 90 days	13 (8.2%)	5 (3.4%)	0.072
Hyponatremia^*^ within 7 days	12 (7.5%)	5 (3.4%)	0.107
Hyponatremia within 30 days	22 (13.8%)	14 (9.4%)	0.225
Hypernatremia^#^ within 7 days	44 (27.7%)	34 (22.8%)	0.328
Hypernatremia within 30 days	56 (35.2%)	42 (28.2%)	0.185
New-onset Liver function injury^∧^ within 7-day	20 (12.6%)	18 (12.1%)	0.894
New-onset Liver function injury within 30-day	36 (22.6%)	27 (18.1%)	0.326

AHF, acute heart failure; CKD, chronic kidney disease.

* Hyponatremia defined as serum sodium < 135 mmol/L;

# Hypernatremia defined as serum sodium > 145 mmol/L; ∧New onset liver function injury defined as alanine aminotransferase (ALT) > 40 U/L or aspartate aminotransferase (AST) > 40 U/L or total bilirubin > 21 μmol/L or direct bilirubin > 8.6 μmol/L, except the history of liver function injury.

## Discussion

In this retrospective cohort study, 716 elderly patients with AHF, including 361 patients with oliguria. The incidence rate of oliguria was 51.9% in all patients with AHF. This study consisted of very elderly patients with a median age of 91 years (88, 95) and a total of 43.2% (133/308) of patients with a history of CKD. The overall mortality at 7 and 90-day of AHF patients with oliguria was 21.8% (67/308) and 60.7% (187/308), respectively. 2021 ESC guidelines for the diagnosis and treatment of HF indicated that ACEIs/ARNIs, β-blockers, mineralocorticoid receptor antagonists (MRAs), sodium-glucose cotransporter 2 (SGLT2) inhibitors (dapagliflozin/empagliflozin) and loop diuretics are the cornerstone treatment for HF (23). Loop diuretics are a first-line standard treatment for AHF and are widely used clinically, and they are usually used at higher doses, especially in patients with oliguria (24). However, diuretic resistance frequently occurs in patients with renal insufficiency, RAAS activation, hypoproteinemia and hyponatremia, especially in elderly patients. Diuretics are usually not sufficient to achieve volume reduction. AHF in elderly patients is usually accompanied by one or more conditions such as RASS activation, renal dysfunction, high urea nitrogen, and acidosis. Hypoalbuminemia is also common in frail and infected elderly patients. High volume loading or anorexia in AHF often causes hyponatremia. Because of these factors, elderly patients with AHF need higher doses of loop diuretics to achieve the same effect, and diuretic resistance comes (25, 26). Diuretic resistance is different from oliguria. The presence of oliguria may mean more severe diuretic resistance, and there is little strategy for treating elderly AHF patients with oliguria. So, in this study, we evaluated not only the efficacy of TLV in diuretic resistant patients with AHF, but also in non-diuretic resistant patients with oliguria with AHF. A total of 43.5% (134/308) of the patients with AHF and oliguria took more than 100 mg of an equivalent dose of furosemide per day. We found that daily use of diuretics in excess of 100 mg furosemide equivalent dose significantly increased the 7 and 90-day all-cause mortality in elderly patients with AHF with oliguria. There was no statistically significant difference in all-cause mortality at 7 and 90-day between the patients who received 100~200 mg furosemide equivalent doses compared to those who received more than 200 mg furosemide equivalent doses of diuretics. In a subgroup analysis, compared with the usage of <100 mg doses of furosemide per day only, the addition of TLV on the basis of < 100 mg doses of furosemide per day had a trend in all-cause mortality reduction at 7-day in older patients with AHF and oliguria, but the difference did not reach statistical significance (Figure 4. E: Group A1 vs. Group B1, *p* = 0.057). However, compared with the usage of 100~200 mg doses of furosemide per day only, TLV addition on the basis of 100~200 mg doses of furosemide per day was associated with a reduction in 7 and 90-day all-cause mortality in elderly patients with AHF and oliguria (Figure 4. E: Group B0 vs. Group B1, *p* = 0.037 and F: Group B0 vs. Group B1, *p* = 0.033). Our study suggests that the addition of TLV on the basis of < 200 mg doses of furosemide may reduce the 7 and 90-day all-cause mortality in elderly patients with AHF and oliguria.

Tanaka et al. (6) reported that the addition of TLV for 7 days in a row but not furosemide significantly increased urine volume, decreased body weight and improved residual congestion in patients with AHF and CKD receiving furosemide treatment. Akihisa et al. (14) also reported that the administration of TLV for 7~14 consecutive days and the decreased use of loop diuretics increased urine volume, alleviated congestion and improved renal function in patients with congestive AHF and renal dysfunction. Yusuke et al. (27) showed that continuous administration of TLV for 6 months may improve long-term adverse outcomes in AHF patients with CKD. Short-term and early administration of TLV could prevent exacerbation of AKI and improve the prognosis for AHF patients within 6 months (16). Continuous treatment with TLV for at least 180 days was also associated with better renal function and good progression because of its ability to spare the dose of loop diuretics for long-term use (13). In the present study, we found that the addition of TLV significantly increased the median urine output in 7 days in elderly patients, but had no significant effect on reducing the NT-proBNP levels and reversing the 7-day AHF status. This confirms the short-term efficacy of TLV addition at increasing urine output and improving AHF status in elderly patients with AHF and oliguria. In terms of mid-term efficacy, we found better results in that the addition of TLV significantly reduced the NT-proBNP levels, and reversed the progression of AHF within 30 days. The lack of effect on improving the AHF status within 7 days may be due to the poor cardiac and renal reserve and the slow response of these reserves to the medications in elderly patients. Within 30 days, the therapeutic effect of TLV gradually appeared.

Some prospective cohort studies showed that there was no strong evidence that proved the mortality benefit of any diuretics, including TLV, in either acute or chronic HF settings (28–30). However, a few *post hoc* analyses showed that TLV usage may be associated with improving HF-related morbidity (31, 32). Although the long-term results of the EVEREST are neutral, the EVEREST *post hoc* analysis in the low sodium subgroup (serum sodium level below 130 mmol/L) showed that the use of tolvaptan had favorable effects on decreasing the cardiovascular mortality and hospitalization rate of HF patients and had no adverse effect on renal function (28). In this retrospective cohort study, we found that the addition of TLV was associated with improving the all-cause mortality at 7 and 90-day in elderly patients with AHF combined with oliguria. This result may be related to the patients we included. In AHF patients, coexisting with oliguria means more severe diuretic resistance and volume overload, which may lead to higher mortality, especially in elderly patients. It also may be associated with the unique mechanism of TLV. When TLV is used in combination with diuretics, urine output could rapidly increase, thus achieving decongestion.

With the occurrence of oliguria, the eGFR level decreased in all AHF patients. However, the proportion of new-onset CKD after 90 days was not high (5.84%, 18/308), and the addition of TLV did not lead to an increase in new-onset CKD after 90 days when the patient's AHF and oliguria were corrected. This is consistent with previous reports (13, 33, 34). Previous studies have reported that the side effects of TLV could increase patient intake, hypernatremia, renal impairment and liver damage (35, 36). In this study, there was also no statistically significant difference between the two groups regarding new-onset CKD after 90 days. Although there was no significant difference in hypernatremia and liver injury between the TLV and non-TLV group, there was still a high incidence of these conditions in the elderly patients. The high incidence of hypernatremia and liver injury may be related to the coexistence of multiple diseases and the decline in organ function in elderly patients. Therefore, we still need to closely monitor the safety of long-term TLV use in elderly patients.

This study has several limitations. First, we could not prove the direct causal relationship between TLV therapy and all-cause mortality in this observational study. Second, this was a retrospective study that was conducted in a single center involving a moderate number of enrolled patients, which were all male and elderly. A possible selection bias could not be excluded. Third, the increase or decrease of TLV and diuretic doses (including administration mode: continuous or bolus) and the administration of other standard treatments for AHF were decided at the discretion of each doctor. Therefore, confounding effects may exist. Fourth, the observation period was relatively short, and a prospective multicenter study with an extended observation period is needed to further determine the efficacy and safety of TLV.

In conclusion, in this study we found that the addition of TLV during the use of loop diuretics was associated with an increase in urine output and may improve the all-cause mortality at 7 and 90-day in elderly patients with AHF coexisting with oliguria.

## Data availability statement

The raw data supporting the conclusions of this article will be made available by the authors, without undue reservation.

## Ethics statement

The studies involving human participants were reviewed and approved by the Medical Ethics Committee of Chinese PLA General Hospital (No. S2022-342-01). Written informed consent was not required in this study as the data was collected anonymously.

## Author contributions

QA and QC conceptualized the study. YL, YZ, HC, ZW, GY, and QA took responsibility for data handling and statistical analysis. YL, YZ, HC, QC, and QA drafted the manuscript. JZ, QM, XW, JH, QC, and QA contributed to the interpretation of the data, critical revision of the manuscript, and study supervision. All authors read and approved the final manuscript.
